# Sex Differences in the Lacrimal Gland: Implications for Dry Eye Disease

**DOI:** 10.3390/ijms26083833

**Published:** 2025-04-18

**Authors:** Snježana Kaštelan, Koraljka Hat, Zora Tomić, Tomislav Matejić, Nikola Gotovac

**Affiliations:** 1 Department of Ophthalmology, Clinical Hospital Dubrava, School of Medicine, University of Zagreb, 10000 Zagreb, Croatia; 2 Department of Maxillofacial Surgery, Clinical Hospital Dubrava, University of Zagreb School of Dental Medicine, 10000 Zagreb, Croatia; 3 Health Centre of the Croatian Department of Internal Affairs, 10000 Zagreb, Croatia; 4 Surgery Clinic, Clinical Hospital Sveti Duh, 10000 Zagreb, Croatia; 5 Department of Clinical Radiology, General Hospital Požega, Faculty of Dental Medicine and Health, J. J. Strossmayer University of Osijek, 31000 Osijek, Croatia

**Keywords:** sexual dimorphism, lacrimal gland, dry eye disease, sex steroid hormones, androgens, oestrogens, tear film, personalised treatment

## Abstract

Sexual dimorphism significantly impacts the lacrimal gland’s structure, function, and ageing processes, playing an important role in dry eye disease (DED) pathophysiology. This multifactorial disorder, characterised by tear film instability, inflammation, and visual impairment, disproportionately affects women, especially after menopause. It highlights the interplay between sex steroid hormones, lacrimal gland function, and environmental factors. Systemic and local androgens are vital for maintaining lacrimal gland health and tear production, while the role of oestrogens remains less clear. Evidence suggests dose and context-dependent effects on inflammation and glandular function. Histopathological and molecular studies reveal significant sex differences in the lacrimal gland, with women exhibiting more pronounced age-related degenerative changes, including fibrosis and acinar atrophy, contributing to their increased susceptibility to DED. Despite these findings, the underlying mechanisms connecting sex steroid hormones, receptor expression, and local tissue regulation to these disparities remain poorly understood, highlighting the need for further research. This review synthesises the current knowledge of sex-specific differences in the lacrimal gland, emphasising the importance of integrating systemic and local biomarkers, histological data, and molecular insights into personalised therapeutic strategies. By tailoring treatments to patients’ unique hormonal and molecular profiles, personalised medicine has the potential to transform DED management, addressing unmet clinical needs and improving outcomes.

## 1. Introduction

Sexual dimorphism is evident across various species, encompassing distinctions between males and females beyond reproductive functions. These differences manifest in secondary sexual characteristics and are observed at genetic, cellular, and systemic levels, profoundly affecting health, disease susceptibility, and life expectancy. The emergence of sex differences is shaped by the interplay of genes, chromosomes, sex steroid hormones (SSHs), and environmental factors, though their precise contributions remain under investigation. The contemporary understanding of the development of sex differences involves a complex interplay of chromosomes, genes, SSHs, and the individual’s environment. However, the precise roles and contributions in this process remain unclear. A thorough understanding of the biology of both sexes and the development of reliable, practical knowledge requires conducting separate studies for each sex [1,2].

Dry eye disease (DED) is a multifactorial disorder affecting the tears and the ocular surface. It results in tear film instability and symptoms such as discomfort and visual disturbances. This condition can potentially damage the ocular surface and is marked by elevated tear film osmolarity and inflammation of the ocular surface [3].

Current research shows that women have a 50–70% higher risk of developing DED compared to men [4], and this risk becomes even more pronounced after menopause, suggesting that female sex and advanced age are significant risk factors for DED [5,6]. As age increases, levels of SSH decline, indicating that systemic and local levels of androgens and oestrogens may play a significant role in the pathophysiology of lacrimal gland dysfunction. Optimal androgen levels for normal lacrimal and Meibomian gland function have been established [7,8,9]. However, the role of oestrogen remains unclear: some studies suggest it has a pro-inflammatory effect, while others point to an anti-inflammatory effect. At the same time, some report no significant impact on the lacrimal gland [10,11,12]. Oestrogens and progestogens most likely play a less prominent role than androgens in driving sex differences in gene expression and lacrimal gland sexual dimorphism. Additional research is necessary for a clearer understanding of their roles and to improve our knowledge of the underlying mechanisms [13,14].

**Figure 1:**
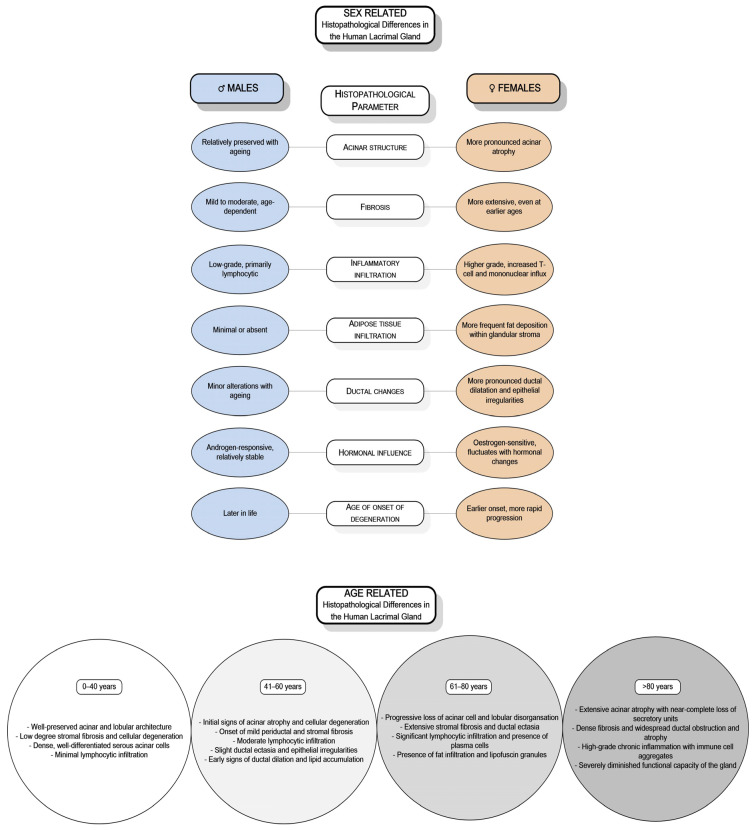
Sex- and age-related histopathological differences of the human lacrimal gland.

DED’s significant public health burden has encouraged extensive research into its genetic, hormonal, biochemical, and molecular foundations [15]. While studies have highlighted sex- and age-related differences in the structure and function of the lacrimal gland, research involving human subjects remains limited, resulting in insufficient data. Only a few investigations have specifically addressed the sex- and age-related differences in the human lacrimal gland [16,17,18,19,20,21,22,23,24]. Observations across various animal models, including mice, rats, and rabbits, have demonstrated sex-based variations in morphology, immunological responses, hormonal regulation, and secretory activity [10,25,26,27,28,29,30,31,32]. Similarly, studies on the human lacrimal gland indicate sex-related differences in age-associated degenerative changes and the expression of SSH receptors [16,17,18,19,20,21,22] (Figure 1).

This review provides a novel and comprehensive perspective on the role of sex differences in the lacrimal gland, encompassing structural characteristics, hormonal regulation, immune responses, and gene expression in the development and progression of DED. While many previous reviews have described DED as a multifactorial disease, few have specifically focused on how biological sex affects lacrimal gland function and contributes to the differences in disease prevalence and severity between sexes. By connecting the findings from experimental models and clinical studies, we aim to highlight how a deeper understanding of sex-based mechanisms may guide the development of more personalised approaches to diagnosis and treatment. This includes the potential use of sex-specific biomarkers for early detection, hormone-targeted therapies, and improved patient stratification in clinical settings.

## 2. The Lacrimal Functional Unit

Tear production involves complex interactions among the nervous, muscular, endocrine, vascular, and immune systems [33]. The lacrimal gland is the primary source of tear film fluid, electrolytes, and proteins. Along with the accessory lacrimal glands, meibomian glands, conjunctiva, cornea, and neural reflex arc, it constitutes the lacrimal functional unit (LFU) [34]. The proper functioning of all LFU components is crucial for efficient ocular lubrication and the maintenance of ocular surface integrity [35]. Disruptions in any part of the LFU, often due to systemic or local inflammation, can lead to DED [34,36].

### 2.1. The Lacrimal Gland

Lacrimal glands are paired exocrine seromucous glands, essential components of the LFU [34], and histologically are classified as tubuloalveolar glands [18]. Tear secretion is a complex, multi-step process. Initially, acinar cells produce a primary fluid, an ultrafiltrate of plasma made of water and ions. As this fluid moves through the canal system, it is altered by ductal epithelial cells incorporating potassium and modifying the protein composition [37]. The final composition of tears includes sodium, potassium, magnesium, calcium, chloride, bicarbonate, and phosphate ions, all of which contribute to tear osmolarity. Ductal cells, which contain numerous secretory granules, play a crucial role in regulating the protein content of tears [33]. The tear film comprises diverse proteins; 491 have been identified, including lipocalin, lipophilin, lactoferrin, lysozyme, serum albumins, and IgA [38,39,40,41].

### 2.2. Tear Film

The tear film acts as a vital barrier, nourishing corneal layers, enabling gas exchange, and supplying nutrients to the avascular cornea. Additionally, the tear film protects the eye’s surface from pathogens, aids wound healing, and enhances eye comfort and visual clarity [40,42]. The quality, quantity, and stability of the tear film are vital for preserving the optical quality, maintaining corneal transparency, and ensuring the integrity of the anterior eye surface [3,43,44]. Disorders affecting components of the LFU, predominantly due to systemic or local inflammation [34,36], often result in DED.

The tear film was traditionally described as having a tri-layered structure consisting of lipid, aqueous, and mucin layers. However, it is now recognised as being more intricately organised. The outer layer is primarily made of lipids along with intercalated proteins. The middle layer contains water, electrolytes, proteins, and mucins. Finally, the inner layer is viscous and connects the tear film to the corneal surface epithelial cells through transmembrane glycoproteins and mucins [45,46,47].

The precorneal tear film acts as a dynamic, integrated unit with distinct layers [48].

The 40 nm thick lipid layer is a critical component of the tear film, playing a vital role in maintaining ocular surface health by reducing evaporation, providing a smooth optical surface, lowering surface tension, preventing tear film collapse, and enabling the tear film to spread evenly across the eye’s surface [43,49,50,51,52,53,54]. Understanding its composition and function is essential for addressing conditions like DED [55]. The lipid layer consists of two main phases: a thin polar phase adjacent to the aqueous layer and a thicker nonpolar phase at the air interface. The polar lipids, such as phospholipids, form a structural base with surfactant properties, while the nonpolar lipids, including wax esters and cholesteryl esters, provide barrier functions [53,56]. Lipids from the meibomian glands play a key role in preventing the collapse of the aqueous layer, with smaller contributions from Zeiss’s and Moll’s glands. Sex hormones, particularly androgens, significantly modulate the lipid profile of the meibomian glands, which are essential for maintaining the tear film’s lipid layer. They primarily act by regulating lipid production and gene expression. Deficiency or dysfunction in androgen signalling can lead to significant meibomian gland dysfunction and contribute to DED [9,57,58,59]. Oestrogen receptors are present in the meibomian glands, indicating that oestrogens also influence these glands’ function, primarily through the modulation of lipid production [60,61,62]. Oestrogen receptors are present in the meibomian glands, indicating that oestrogens also influence these glands’ function, primarily through the modulation of lipid production. The effects of oestrogen on lipid layer formation remain controversial. Most studies suggest that oestrogens promote inflammation, decrease secretion from the meibomian glands, and inhibit lipogenesis, potentially contributing to DED symptoms, especially in postmenopausal women [63,64,65,66], while others suggest a protective role [67]. However, the direct impact of these hormonal changes on the stability and function of the lipid layer of the tear film remains unclear and warrants further investigation.

The aqueous and mucin layers together form a gel-like mucoaqueous layer. Most of the aqueous component is secreted by the lacrimal gland, with approximately 10% originating from accessory lacrimal glands [68,69]. Corneal and conjunctival epithelium also contribute to the aqueous component by secreting electrolytes, water, and mucins [33,34,70].

## 3. Dry Eye Disease

Dry eye disease, known as keratoconjunctivitis sicca, is a multifactorial condition characterised by instability and tear film deficiency. This leads to discomfort and visual impairment and is often associated with varying degrees of ocular surface epitheliopathy, inflammation, and neurosensory abnormalities [71,72,73]. The key diagnostic criteria for DED include tear film instability, inflammation, ocular discomfort, and visual impairment [73,74].

The pathophysiology of DED primarily involves evaporative water loss, which leads to hyperosmolar damage to the ocular tissues. This damage results in the loss of epithelial and goblet cells, directly or through induced inflammation [75]. The reduction in ocular surface moisture accelerates the tear film’s rupture and further increases hyperosmolarity, creating a vicious cycle [43,74]. Persistent inflammation and epithelial breakdown expose nociceptive receptors on the eye’s surface, triggering sensory nerve activation and causing discomfort. Additionally, the instability of the tear film disrupts its optical properties, contributing to visual impairment [44,73,74,75,76,77,78,79].

Mechanical abrasions due to lid margin disorders, such as the obstruction of meibomian gland openings and conjunctivochalasis, can cause micro-trauma of the ocular surface during blinking and affect tear dynamics. Eyelid abnormalities, including lagophthalmos, incomplete or reduced blinking, and poor eyelid-to-eye adhesion, can further contribute to tear film instability [74].

### 3.1. Classification of Dry Eye Disease

Dry eye disease is characterised by inadequate production or excessive evaporation of tears, leading to altered tear osmolarity and increased osmotic stress on the ocular surface [72,73]. DED can be categorised into two primary subtypes based on aetiology: aqueous tear-deficient dry eye and lipid-layer-deficient evaporative dry eye. It is not uncommon for patients to display features of both subtypes simultaneously [77]. Typically, meibomian gland dysfunction is associated with evaporative dry eye, while lacrimal gland dysfunction primarily relates to aqueous tear-deficient dry eye. It is now widely accepted that meibomian gland dysfunction can be classified into two categories based on gland secretion: the hyposecretory (or obstructive) subtype and the hypersecretory subtype [78]. More broadly, DED can be categorised into four subtypes: (1) lipid-deficient dry eye, resulting from abnormalities in the tear film’s lipid layer; (2) aqueous-deficient dry eye, characterised by insufficient tear production, including cases of primary mucin deficiency; (3) dry eye induced by allergies or environmental irritants; and (4) dry eye associated with eyelid surface anomalies, including primary epitheliopathy and structural abnormalities of the eyelids. Dry eye with a lipid anomaly and dry eye with aqueous tear deficiency are the most common subcategories [62].

### 3.2. Prevalence of Dry Eye Disease

The prevalence of dry eye symptoms and tear film dysfunction increases with age, affecting between 5% and 34% of the population, depending on the diagnostic criteria and demographic factors [80]. In the United States, 6.8% of the adult population meets the diagnostic criteria for DED. The condition is notably more prevalent among older adults, with 2.7% of those aged 18–34 affected compared to 18.6% of those over 75. Furthermore, women are more frequently affected than men, with 8.8% of women and 4.5% of men experiencing dry eye symptoms [81].

Albietz et al. reported a dry eye prevalence of approximately 10.8%, with notably higher rates in individuals over the age of 40 (18.1%) compared to those under 40 (7.3%) [82]. The study identified lipid-anomaly dry eye as the most common subtype, occurring in 4.0% of the population, followed by allergic and toxic dry eye at 3.1%, lid-surfacing anomalies dry eye at 1.8%, and aqueous tear deficiencies at 1.7%. Notably, aqueous tear deficiencies exhibited a significant gender disparity, being more prevalent in women. Lipid-anomaly dry eye and aqueous tear deficiencies were significantly more common in individuals over 40 [79]. Since women face a 50–70% higher risk of developing DED, with the risk increasing further after menopause [4], both female gender and ageing are key risk factors for DED [4,36,79].

### 3.3. Risk Factors and Pathophysiology of Dry Eye Disease

**Figure 2:**
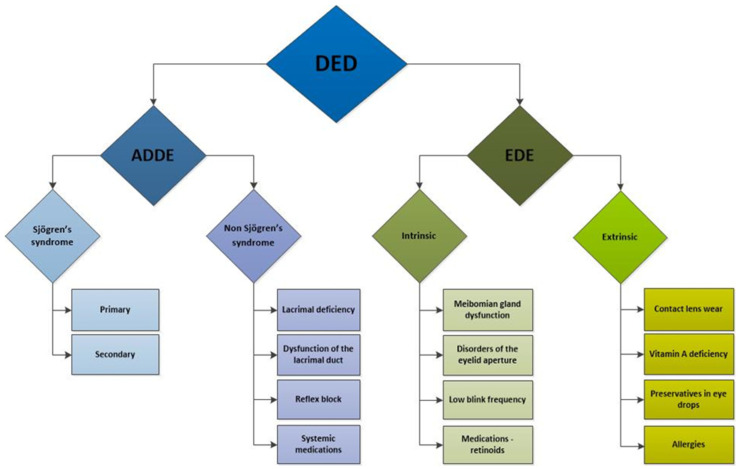
Schematic presentation of dry eye disease subtypes and etiological factors contributing to its development. DED: dry eye disease; ADDE: aqueous tear-deficient dry eye; EDE: evaporative dry eye.

Numerous factors influencing the development and progression of dry eye have been identified and can be categorised into intrinsic and extrinsic factors [83] (Figure 2). Intrinsic factors include autoimmune disorders [83,84,85,86,87,88], hormonal imbalances [75,89,90,91], systemic diseases such as diabetes mellitus [92,93], hereditary disorders [94,95], nerve damage [96,97], and intestinal dysbiosis [98,99].

Extrinsic factors encompass environmental influences affecting lacrimal function [100,101]. These include behaviours and habits such as smoking and excessive screen time [102,103,104,105], contact lens wearing [106], laser eye surgery [107,108,109], the use of certain systemic and ocular medications such as antidepressants, antipsychotics, beta-blockers, diuretics, oral contraceptives, and topical beta-blockers for glaucoma [110], and diets low in omega-3 fatty acids [111,112].

The most prevalent autoimmune disorder associated with DED is Sjögren’s syndrome, which primarily affects the lacrimal and salivary glands and has a significant female predominance, with over 90% of patients being women [93]. Other autoimmune disorders, such as sarcoidosis and Graves’ disease, can also cause non-infectious dacryoadenitis and DED. Less commonly, dry eye may arise from other chronic inflammatory conditions, including IgG4-related disease, inflammatory orbital pseudotumor, chronic graft-versus-host disease, and diabetes, as well as from radiation and infections such as HIV, CMV, and hepatitis C [3,111].

Emerging evidence underscores the significant role of chronic inflammation in DED. Research indicates that chronic inflammation and autoimmunity are primary etiological factors contributing to DED. Elevated levels of inflammatory mediators, including IL-1β, IL-6, IL-8, TNF-α, IL-17, and IFN-γ, have been found in the conjunctiva and tear fluid of patients with dry eye compared to healthy controls [9,112,113]. Furthermore, DED is associated with several pathological changes, including an increased production of matrix metalloproteinases, heightened levels of chemokines and oxidative stress markers, and exacerbated squamous metaplasia of the ocular surface epithelium. These changes are accompanied by a loss of goblet cells and increased endoplasmic reticulum stress [40,114].

With ageing, the immune system becomes dysregulated, leading to changes in antibodies and cytokines and a decline in immunity, with an increase in autoimmunity. The lacrimal gland also undergoes functional changes, such as reduced innervation and secretory activity [115]. Structural changes include atrophy of acini and fibrosis, periductal fibrosis, ductal dilation and proliferation, lymphocytic infiltration, and fatty infiltration [17]. These structural changes correlate with a decline in the gland’s secretory function [17,33].

## 4. The Role of Sex Steroid Hormones in the Development of Sex Differences in Dry Eye Disease

SSHs, particularly androgens and oestrogens, significantly influence the structure and function of the human lacrimal gland, which is crucial for tear production and ocular health. These hormones interact with specific receptors in the lacrimal gland, affecting its physiology and potentially contributing to conditions like dry eye disease. Sex differences mainly develop throughout complex interactions of SSHs at the local and systemic levels, such as receptor expression and gene regulation, but this complex mechanism has not been elucidated [1,2,6,9,23,57,59,62].

SSHs are a subset of steroid hormones derived from cholesterol. They play a crucial role in regulating various physiological processes related to sexual development and reproduction. In humans, SSHs are classified into three main groups: androgens, oestrogens, and gestagens [13,116,117,118]. Androgens, often classified as male hormones, are present in both males and females. Key androgens include dehydroepiandrosterone sulphate, dehydroepiandrosterone, androstenedione, testosterone, and dihydrotestosterone. Oestrogens, primarily known as female hormones, also have a significant role in males. Key oestrogens include estrone, 17-β-estradiol, and estriol. Gestagens are involved in regulating the menstrual cycle and maintaining pregnancy. Key gestagens are progesterone and 17α-hydroxyprogesterone. Each of these hormones has specific functions, but they often interact with each other to maintain hormonal balance and support various bodily functions [116,117,118].

### 4.1. Synthesis of Sex Steroid Hormones

The synthesis of SSHs in the human body shows gender-specific patterns, including differences in hormone levels, predominant types, and secretion rhythms. Women primarily produce β-estradiol, whereas men have higher testosterone levels.

Steroidogenesis is the process by which cholesterol is converted into biologically active steroid hormones. This conversion occurs in the gonads, adrenal cortex, and various tissues throughout the body. The regulatory control of steroidogenesis is exerted by trophic hormones from the anterior lobe of the pituitary gland, namely follicle-stimulating hormone, luteinising hormone, and adrenocorticotropic hormone. Within the target cell, steroidogenesis is facilitated by enzymes found in the mitochondria and the smooth endoplasmic reticulum.

**Figure 3:**
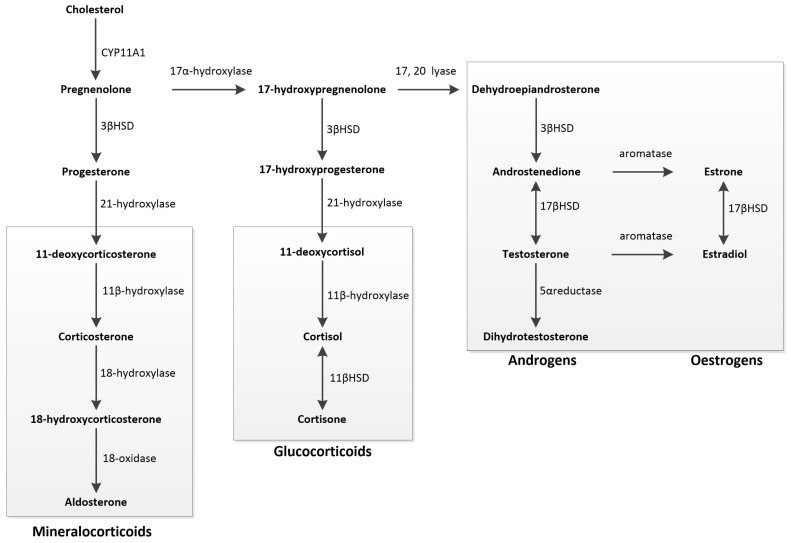
Schematic overview of steroidogenesis: Steroidogenesis begins with cholesterol, converted into pregnenolone, the precursor for all mineralocorticoids, glucocorticoids, and sex steroids. Pregnenolone can be metabolised into either progesterone or 17-hydroxypregnenolone. Progesterone serves as the primary precursor for mineralocorticoids. 17-hydroxypregnenolone acts as the precursor for all androgens, oestrogens, and glucocorticoids. Androgens can undergo aromatisation to form oestrogens, while testosterone can be converted into dihydrotestosterone through 5α-reductase action.

The initial step involves the transfer of cholesterol to the inner mitochondrial membrane, a process regulated by the steroidogenic acute regulatory protein. Cholesterol is then converted into pregnenolone, the precursor for all mineralocorticoids, glucocorticoids, and SSHs, through the action of the cytochrome P450 SCC enzyme CYP11A1. Pregnenolone is converted into either progesterone or 17-hydroxypregnenolone. Progesterone is a mineralocorticoid precursor, while 17-hydroxypregnenolone is a precursor for androgens, oestrogens, and glucocorticoids. Oestrogens are produced from androgens through aromatisation, and dihydrotestosterone is formed from testosterone by the action of 5α-reductase [119] (Figure 3).

In the testicles, Leydig cells, located in the interstitium between seminiferous tubules, predominantly synthesise testosterone [120]. SSHs are synthesised in small amounts in the third layer (zona reticularis) within the adrenal gland cortex, with androstenedione being the primary product. In the ovary, granulosa cells predominantly produce progesterone and 17-β-estradiol, while theca cells produce androgens, and luteal cells produce progesterone [119].

Recent studies have demonstrated that various tissues express enzymes capable of activating steroid precursors, a process known as extra-glandular steroid activation, and synthesising active steroids de novo through a mechanism of extracrine steroidogenesis. As a result, many tissues and organs previously considered non-steroidogenic are now recognised for their ability to biosynthesise steroids. Local steroid hormone synthesis is well-established in the brain [121,122], spinal cord [123], and peripheral nerves [124], where these hormones are collectively referred to as neurosteroids [124,125,126,127]. Moreover, the extra-glandular steroidogenesis of SSHs has been identified in various tissues, including adipose tissue [128], skin [129], exocrine glands [130], the small intestine [131], and the kidneys [130]. Furthermore, the local synthesis of glucocorticoids has been documented in the intestinal mucosa [126,130] and thymus [127,132,133,134,135,136,137]. SSHs, along with genetic and environmental factors, play a crucial role in the development of sexual differences. The biological significance of extra-glandular steroidogenesis is associated with paracrine, autocrine, or intracrine signalling being mediated by these locally produced molecules [122]. Furthermore, the synthesis of these steroids is often regulated by complex multifactorial systems independent of hypothalamic and pituitary control. Therefore, measuring local steroid levels is frequently a more precise indicator of steroid action within specific tissues when compared to systemic levels [123].

### 4.2. Steroid Hormone Receptors

Steroid hormone receptors belong to the nuclear receptor superfamily and function as ligand-dependent transcription factors [124]. This superfamily encompasses androgen receptor (AR), oestrogen receptor (ER), progesterone receptor (PR), glucocorticoid, and mineralocorticoid receptors [128,133], in addition to retinoic acid and thyroid hormone receptors [124].

The role of SSHs in shaping sexual differences is intricate, as research has shown that specific hormones exert sexually distinct, tissue-specific, and cell-specific effects [138,139,140]. SSHs and genetic and environmental factors are essential in developing gender differences. mRNA for AR, ER, and PR is present in various ocular tissues, including the lacrimal and meibomian glands, eyelids, palpebral and bulbar conjunctiva, cornea, iris, ciliary body, lens, retina, choroid, and retinal pigment epithelial cells in rats, rabbits, and humans [140,141,142,143].

It is recognised that sex-related differences in the prevalence of DED are predominantly linked to the influences of SSHs, the hypothalamic–pituitary axis, corticosteroids, insulin, IGF-1, pineal hormones, along with sex chromosomes, sex-specific autosomal factors, and epigenetic factors [4]. As ageing causes a reduction in SSH levels, both androgen and oestrogen become vital in the pathophysiology of lacrimal gland dysfunction. Importantly, androgens are significant, and their deficiency is connected with dysfunction in the meibomian and lacrimal glands, which contributes to DED [7,8,9,13,63,142,143].

Experimental studies involving castration or androgen antagonist exposure in mice have shown considerable changes in the anatomy and physiology of the lacrimal gland. These changes include degenerative alterations such as reduced growth, loss of glandular tissue, diminished size of acini and nuclei, nuclear polymorphism, increased connective tissue proliferation, disruptions in protein levels, changes in enzyme activity, modifications in fluid and protein secretion, and altered gland morphology, leading to feminisation of the male gland [142].

### 4.3. Androgens

Androgens regulate the expression of over 2200 genes in the lacrimal gland of mice and approximately 3000 genes in the human meibomian gland and conjunctival epithelial cells [28,144]. These genes are involved in various functions, including cell growth, proliferation, metabolism, cell communication and transport, nucleic acid binding, signal transduction, and receptor activity. Thus, androgens exert a multi-faceted influence on the structure and function of the lacrimal gland at multiple levels [7,10,25,29,145,146,147]. Androgen deficiency has also been noted in women with Sjögren’s syndrome, suggesting that androgens may facilitate disease progression rather than cause it directly [141,148].

Androgen receptors have been identified in acinar and epithelial cells of the lacrimal ducts [26,149]. Androgens also affect the expression of their receptors in the lacrimal gland, increasing androgen receptor protein levels while decreasing androgen receptor mRNA levels. In a mouse model, androgen administration significantly increases the number of cells containing androgen receptors and the density of these receptors within the nuclei of epithelial cells in the lacrimal gland. This regulatory effect is specific to androgens, as similar effects are not observed with oestrogen, glucocorticoids, or cyclophosphamide. Importantly, cessation of androgen therapy leads to a substantial decrease in AR expression [26,150,151,152].

### 4.4. Oestrogens

Previous research on the effects of oestrogen on the lacrimal glands has yielded inconsistent results [4,13,27,29,153]. Hormone replacement therapy, including oestrogen and medroxyprogesterone acetate, has been shown to enhance tear production, although it does not necessarily improve tear quality [154,155]. Reduced lacrimal gland function has been noted in postmenopausal or ovariectomised women, as well as in women using oral contraceptives, despite fluctuations in oestrogen levels [156,157].

Oestrogen has demonstrated an anti-inflammatory effect in mouse models of Sjögren’s syndrome, which contrasts with the findings from studies showing that hormone replacement therapy in postmenopausal women is associated with an increased risk of DED [11]. This discrepancy may arise from the differential effects of high versus low doses of oestrogen on inflammation [10,11]. Research suggests that lower doses of oestrogen can promote cell survival and provide protective effects against inflammation in exocrine glands, whereas higher doses may exacerbate inflammatory responses [12,158,159,160].

Recent research suggests that oestrogen and progesterone have a relatively minor influence on sex-related differences in gene expression and structural variations in the lacrimal gland compared to androgens [161,162]. However, Hat et al., in their analysis of 35 human lacrimal gland tissue samples from 19 cornea donors, observed significantly higher ERα mRNA expression than AR and ERβ [163]. Furthermore, antiandrogenic therapy in men does not appear to affect tear secretion, emphasising a sex-specific role of androgens in lacrimal gland function. While testosterone and dihydrotestosterone have shown efficacy in rat models, the current evidence is insufficient to justify their use in human clinical trials [164]. The presence of mRNAs for steroidogenic enzymes in the human lacrimal gland indicates the potential for intracrine synthesis and metabolism of sex steroids, offering valuable insights into the complex relationship between systemic sex hormone levels and DED [130].

## 5. Sexual Dimorphism and the Development of Sexual Differences

Sexual dimorphism is any structural difference between male and female individuals of the same species [165], excluding differences directly related to reproduction. It broadly encompasses sex differences across the genome, transcriptome, proteome, metabolome, and phenotype [166]. Most sexually dimorphic traits evolved through sexual selection to enhance reproductive success, with a smaller proportion shaped by natural selection [167,168]. Natural, sexual, and environmental selections interact as individuals and are influenced by their surroundings [169,170]. In sexually reproducing species, differences between males and females typically reflect sexual dimorphism, ranging from subtle to pronounced depending on the species.

**Figure 4:**
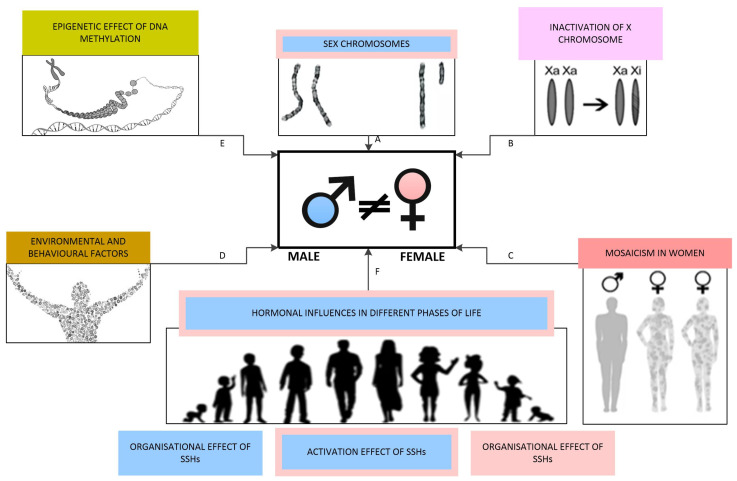
Factors influencing the development of sex differences: (**A**) Sex chromosomes determine chromosomal sex, with the Y chromosome facilitating male gonadal differentiation through the *sex-determining region Y gene*. (**B**) Different extents of X chromosome inactivation contribute to sex differences. (**C**) Mosaicism occurs in various tissues in females. (**D**) Environmental and behavioural factors directly impact sex steroid hormone receptors as endocrine disruptors and induce epigenetic changes. (**E**) Epigenetic influences are primarily mediated through DNA methylation. (**F**) Levels of SSHs at various life stages play a crucial role in shaping and enhancing sex differences.

In mammals, sex is categorised as binary, distinguishing between male and female individuals. The process of sex differentiation involves a series of events during foetal development that shape an individual’s sex, including the formation of gonads and the secretion of sex hormones. This process is sequentially divided into three phases: the determination of chromosomal sex at fertilisation, the establishment of gonadal sex, and the development of primary sexual characteristics. Postnatally, the deepening of sex differences is mainly influenced by sex hormone levels and environmental and behavioural factors, with the most pronounced changes occurring during puberty [116,171]. The factors impacting the development of sex differences are schematically illustrated in Figure 4.

## 6. Sexual Dimorphism of the Lacrimal Gland

The public health burden of DED prompted numerous studies focused on the genetic, hormonal, pathophysiological, biochemical, and molecular basis of this disorder [15]. In the mid-20th century, interest in the histological features of the lacrimal gland increased significantly due to its crucial role in maintaining ocular surface homeostasis. Various animal models have been developed to elucidate the physiology and pathophysiology of lacrimal gland dysfunction and its contribution to DED. As a result, most of the current research has been conducted using these animal models [10,23,24,32,172]. Studies involving human subjects are comparatively rare [16,17,149,163,173,174,175,176].

### 6.1. Sexual Dimorphism of the Lacrimal Gland in Animal Models

Previous research has confirmed the presence of sex differences across various mammalian species. However, interpreting these results requires careful consideration of the structural differences in the lacrimal glands among different laboratory animals [173,177,178], such as mice, rats, rabbits, and hamsters. Most research on animal models highlights substantial structural changes in the lacrimal gland, including inflammatory infiltration, loss of innervation, and the progression of degenerative changes in glandular tissue with age. These changes lead to functional impacts on the quantity and composition of tears [177].

The most commonly observed structural alterations include acinar degeneration, connective tissue proliferation, periductal and periacinar fibrosis, lymphocytic infiltration, proliferation of excretory ducts, and ductal dilation, with thinning of their walls [179,180]. Research indicates that acini undergo a progressive transition with age, shifting from serous to seromucous and eventually to mucous acini, which leads to decreased protein and increased mucus production [181]. These structural alterations in the lacrimal glands of older rats have been linked to reduced tear production, lower protein content in tears, and changes in the corneal epithelium [16,142,176,180,182,183,184]. In the rat model, female lacrimal glands have a smaller surface area but higher acinar density when compared with male glands, with more pronounced differences in older age groups [180]. Similar findings were observed in prostaglandin receptor knockout mice (PRLR -/-) [185], suggesting more significant acinar atrophy in females. In older female samples, periacinar fibrosis was markedly more pronounced [180]. Additionally, females exhibited more extensive proliferation of excretory ducts, while males showed more significant lymphocytic infiltration and females more mast cell infiltration [180,185].

Sex, SSHs, and environmental factors are recognised as key regulators of the ocular surface and adnexal tissues, influencing the prevalence of DED between sexes [4]. Beyond the well-documented physiological differences in SSH levels between males and females, research has revealed sex-specific variations in the regulation of gene expression activated by these hormones. For instance, sex differences in AR expression have been identified in rat lacrimal glands [150]. In ovariectomised mice, testosterone, estradiol, and progesterone regulate the expression of thousands of genes in the lacrimal and meibomian glands. Although many genes regulated by testosterone in female tissues are similar to those in males, distinct sex-specific differences in gene activation have been observed. In some cases, androgens have even opposite effects on the same gene depending on the sex, highlighting how these sex differences in gene regulation contribute to sexual dimorphism [28,32,180].

While current research confirms the presence of sex differences in various mammalian species, it is essential to consider the differences in lacrimal gland structure across different species of laboratory animals when interpreting the results [173,186,187,188]. Studies using various animal models have significantly advanced our understanding of the molecular, endocrine, and immunological mechanisms underlying sex-related differences in the structure and function of the lacrimal gland [25,27,29,31,139,147,172,182]. However, species-specific anatomical and physiological characteristics limit the direct translation of these findings to human biology. Bridging this gap requires validation through human-focused research to ensure accurate interpretation and clinical relevance of the sex-based differences observed in preclinical studies.

### 6.2. Sexual Dimorphism of the Human Lacrimal Gland

Sex differences have been observed in various ocular structures, including the meibomian gland, lacrimal gland, conjunctiva, cornea, anterior chamber, iris, ciliary body, lens, vitreous body, retina, nasolacrimal duct, and tear film. The influence of SSHs on these structures has also been documented [4,16,152,179,181,183,184,187,189,190,191]. Research on human eye tissues and the lacrimal apparatus has confirmed sex differences in tissue morphology, gene expression, protein and lipid synthesis, secretory activity, immune function, cell density, epithelial dynamics, permeability, immune response, tear film stability, blink rate, and visual acuity [4,26,28]. These sex-related differences play a significant role in the onset and progression of various eye diseases, such as DED, refractive errors, myopia, glaucoma, cataracts, age-related macular degeneration, diabetic retinopathy, vernal keratoconjunctivitis, impaired vision, and blindness [192,193,194,195,196,197].

A limited number of studies on human lacrimal gland tissue have primarily focused on age-related pathohistological changes, with only a few addressing sex differences (Table 1). The most frequently observed pathohistological changes include reductions in gland weight, fibrosis, atrophy of acini, alterations in ductal structures, and lymphocytic infiltration [6,16,17,19,20,21,23,149,157,173,174,175,176,185,198,199].

Prager A. 1966 first described senile alterations in the lacrimal gland, noting reductions in gland weight, fibrosis, and acinar atrophy [176]. Similar age-related changes were also reported in a study of 99 cadaveric glands conducted by Damato et al. [173]. They identified several key changes: acinar atrophy, excretory duct obstruction, and periductal fibrosis, accompanied by the infiltration of lymphocytes and polymorphonuclear cells. They also observed mild vasculitis of the periductal blood vessels, which could contribute to chronic inflammation in this region. Their findings suggest that acinar atrophy and fibrosis can begin before middle age but become significantly more pronounced with advancing age [173]. These findings highlight the importance of ductal changes, proposing that repeated episodes of subclinical inflammation over a lifetime may lead to obstruction of the excretory ducts. This obstruction can result in periductal fibrosis that progressively extends into the lobules. The resulting obstruction and subsequent dilation and tortuosity of the ducts, sometimes reaching the point of cystic formation, can lead to variability in the acinar atrophy among different lobules [173].

Supporting these observations from Damato et al. [173], Roen JL and colleagues [185] studied 32 cadaveric lacrimal glands. They found that 75% exhibited microscopic abnormalities, with chronic inflammation and periductal fibrosis being the most common histopathological features. Notably, 52% of glands from individuals over 50 showed periductal fibrosis, while 74% exhibited ductal changes.

While current research has provided valuable insights into age-related changes in the lacrimal gland, research on sex differences in the human lacrimal gland regarding structure and function remains limited. Until now, few studies have investigated the sex differences in the human lacrimal gland. In 1963, Waterhouse reported a higher incidence of focal adenitis in the female lacrimal glands [19]. The first systematic investigation into sex differences in the structure of the human lacrimal gland was published by Cornell-Bell and colleagues in 1985 [16]. Using stereology software, they measured the surface area of 50 acini, revealing a 21% difference between sexes, with average acinus areas of 2.18 mm^2^ in men and 1.80 mm^2^ in women. This study was conducted on a sample of five glands per sex. Additionally, they measured acinar diameter in a lacrimal gland in rats, mice, guinea pigs, and rabbits, identifying significant interspecies differences, suggesting a potential correlation between sex differences and androgen levels [16].

The most comprehensive investigation of the pathohistological features of the human lacrimal gland and sex-related differences was conducted by Obata et al. [17]. They analysed 80 donor cadaveric lacrimal glands and thoroughly examined eight pathohistological features: fibrosis, acinar atrophy, periductal fibrosis, interlobular ductal dilation, interlobular ductal proliferation, lymphocytic foci, periductal lymphocytic infiltration, and fatty infiltration in both the orbital and palpebral lobes. The study found a statistically significant higher prevalence of diffuse fibrosis and diffuse acinar atrophy in women over 60. The findings suggest notable sex differences in pathohistological features and propose a potential link between specific pathohistological changes and DED [17]. Additionally, research into sex differences in lacrimal gland weight has indicated that male glands tend to have greater absolute weight [20]. A magnetic resonance imaging study showed that the thickness and area of the lacrimal gland decreased with age in women but not in men [200].

In a recent study, Hat et al. [174] investigated the pathohistological features of the ageing human lacrimal gland and found notable differences based on sex. Their research demonstrated that the connective tissue and fat volume density within the lacrimal gland of female subjects increases with age. In contrast, no significant correlation between age and these parameters was observed in male subjects, suggesting that age-related connective and fatty degeneration is more pronounced in women. The study also highlighted that, in males, intralobular fibrosis becomes more prominent with age, as indicated by a significant increase in the volume density of intralobular connective tissue. However, the volume density of intralobular adipose tissue did not exhibit a statistically significant correlation with age in either gender, indicating it is not a major factor in age-related degenerative changes [174]. According to the research by Hat et al. [174], periacinar fibrosis does not seem to depend on age or sex, which contrasts with the findings by Obata et al. [17], who found a significant positive correlation between acinar atrophy and age in both sexes, with a notably higher prevalence in women. The predominance of interlobular ductal dilation in the palpebral lobes suggests obstruction of tear outflow in the conjunctival fornix [17]. In contrast, Hat et al. [174] reported a significantly higher frequency of ductal dilation in male glands, probably caused by the unequal representation of palpebral and orbital lobes in their samples. Additionally, their study observed lymphocytic infiltration in 69% of subjects, with no gender differences, which is consistent with earlier findings [173,175].

It is believed that chromosomes, genes, and SSHs are key factors driving the development of sex differences. However, their specific contributions to the structure and function of the lacrimal gland remain incompletely understood. The impact of SSHs on the human lacrimal gland is particularly noteworthy due to its role in the development of DED [142,185]. Despite the established presence of SSH receptors in various eye tissues [196], research on this subject is limited [23,150,197].

Previous studies by Smith et al. [149] and Rocha et al. [6] reported the immunohistochemical identification of androgen receptor protein in the human lacrimal gland. On the other hand, Rocha et al. found AR in two human lacrimal gland samples with mRNA for 5α-reductase, suggesting local regulation of androgen levels in the lacrimal gland [6]. Further, two studies have reported immunohistochemical detection of ER in the human lacrimal gland. One study showed weak staining in a small number of acinus cells in 12 out of 20 cadaveric lacrimal gland samples; another study detected ERβ in 9 out of 10 samples and a weak ERα signal outside the cell nucleus in 2 out of 10 samples [198].

Wickham et al. detected androgen, oestrogen, and progesterone receptor mRNA expression in a sample from three human lacrimal glands. However, their study did not quantify the results or examine the differences between the sexes. Additionally, their research found mRNA for these receptors in lacrimal gland acinar epithelial cells, meibomian glands, the eyelid and bulbar conjunctiva, the cornea, the iris/ciliary body, the lens, the retina/uvea, and retinal pigment cells in rat, rabbits, and human samples [23]. Similarly, Spelsberg et al. detected mRNA for ERα and ERβ in several ocular tissues, including the lacrimal gland, using samples from ten donor lacrimal glands but did not quantify the results or investigate sex differences [199].

The first study to quantify the mRNA expression of SSH receptors (AR, ERα, and ERβ) in the human lacrimal gland was conducted by Hat et al. [163]. This study not only quantified and compared the relative mRNA expression of these receptors but also performed immunohistochemical verification and quantification of the corresponding proteins, offering new insights into the complex factors contributing to sex differences in the human lacrimal gland. This study confirmed the presence of AR, ERα, and ERβ mRNA in the human lacrimal gland using quantitative polymerase chain reaction and, for the first time, quantified their relative mRNA expression levels. It was found that the relative mRNA expression of ERα was significantly higher than that of AR and ERβ. No sex-related differences were observed in the relative mRNA expression of these sex hormone receptors, nor was there any correlation between mRNA expression and age. Immunohistochemical analysis further demonstrated that the protein expression levels of ERα and AR corresponded with their mRNA expression levels [163], suggesting more complex mechanisms of regulation of SSH receptor expression.

## 7. Perspective and Conclusions

Many sex differences emerge from hormonal influences and genetic variations between the sexes. Advances in molecular biology have highlighted that “every cell has a gender”, underscoring the importance of studying sex differences at the cellular and molecular levels. This approach enhances our understanding of how genes drive biological organisation across cells, organs, and organ systems. Furthermore, it sheds light on the roles of sex chromosomes, hormones, and epigenetic mechanisms in shaping physiological differences and their implications for health and disease.

The lacrimal gland has garnered increasing research interest over recent decades due to its critical role in maintaining tear film homeostasis. This attention has led to the development of animal models to explore its physiology, pathophysiology, and contributions to DED [23,24,172,201].

Despite this growing interest, research on human lacrimal glands remains sparse, leaving many questions unanswered. Existing studies have confirmed sex-related differences in the ocular tissues’ morphology, physiology, and pathology, including the lacrimal gland. These differences are primarily shaped by sex chromosomes, genes, SSHs, and environmental factors [10,14,16,17]. However, sex differences in this area often receive insufficient attention.

While it is evident that sex differences arise from a complex interplay of genetic, hormonal, and environmental factors, the specific contributions of each remain unclear [116]. Most documented differences are linked to gene expression variations, heavily influenced by SSHs [29,32,180]. Studies have shown that sex chromosomes alone cannot induce sex differences in the lacrimal glands without the influence of SSHs [31]. Sex significantly affects gene expression in the lacrimal glands, with females showing an increased expression of the genes related to inflammation and immune responses in certain autoimmune conditions like Sjögren syndrome. Over 490 genes in the mouse lacrimal gland show sex-related differential expression, affecting various biological processes and molecular functions [29,32]. The presence of androgen and oestrogen receptors in the lacrimal gland suggests a genetic basis for the hormonal regulation of these differences [163,202]. In the absence of sex hormones, as seen in SF-1 knockout mice, sexual dimorphism in the lacrimal gland is absent, indicating that sex chromosomes alone cannot induce sex differences in lacrimal glands without the influence of SSHs [31]. Additionally, studies on animal models, including ovariectomised and orchiectomised rats, have shown that although androgen receptor expression levels are similar in both sexes, intact male rats exhibit significantly higher androgen receptor levels. This indicates that androgens can independently regulate their receptor expression [7,8]. Additionally, imbalances in steroid hormone levels, either in excess or deficiency, modulate receptor activity through up- and down-regulation processes [203].

In men, serum testosterone levels naturally decline between the ages of 35 and 40, decreasing by approximately 0.5–2% annually [204]. Significant reductions are typically observed in older age, with about 20% of men over 60 and 50% over 80 experiencing testosterone levels below the normal range for younger men [205]. In women, testosterone levels decrease from the fourth decade of life, falling to about 50% of premenopausal levels by menopause and further decreasing over the subsequent 2–5 years [206]. Daily, cyclical, and seasonal fluctuations in serum SSH levels, alongside various physiological and psychological factors, add complexity to hormonal regulation [196]. Individual variations in SSH receptor expression within ocular tissues further complicate this dynamic [196]. The presence of enzymes for steroid synthesis in the lacrimal gland suggests a role for intracrine signalling in modulating its function. These complexities underscore the intricate relationship between systemic SSH levels and local receptor expression, with no apparent differences between sexes [130,206].

Research indicates that SSH receptor expression alone does not fully account for the observed sex differences in the human lacrimal gland. Most studies have found no significant differences in the relative mRNA expression levels of AR, ERα, or ERβ between sexes. This suggests that additional factors play pivotal roles in shaping sex differences in the lacrimal gland.

Future studies on sex differences in the lacrimal gland should address the current limitations by involving living participants of all ages and sexes. Such studies should integrate systemic and local biomarkers, including SSH levels, receptor expression, and tear film quality, with histological evaluations of lacrimal gland tissues. This comprehensive approach could illuminate how sex differences influence glandular function and contribute to DED. Furthermore, it could pave the way for personalised therapeutic strategies to improve outcomes for individuals affected by this condition.

In conclusion, this review highlights the crucial yet often overlooked role of sexual dimorphism in lacrimal gland biology as a key factor in the pathophysiology of dry eye disease. Integrating sex-specific molecular profiles, hormone receptor expression patterns, and immune response characteristics from experimental and clinical studies highlights the urgent need to move beyond conventional “one-size-fits-all” approaches to DED management. A key innovation proposed by this synthesis lies in identifying sex-informed diagnostic and prognostic biomarkers, such as androgen-responsive tear proteins, sex-specific inflammatory mediators, and lacrimal gland morphometric parameters that may enhance early detection and disease monitoring. Furthermore, therapeutic strategies targeting hormonal pathways, including selective androgen receptor modulators and oestrogen antagonists or strategies aimed at modulating sex-biased immune responses, represent promising avenues for future research and clinical application. Recognising and leveraging these sex-based differences not only deepens our understanding of DED pathogenesis but also paves the way for more personalised and effective ocular surface therapies. Ultimately, incorporating sex as a fundamental biological variable in both basic and translational research is essential to improving clinical outcomes and optimising therapeutic efficacy for individuals affected by DED.

## Figures and Tables

**Table 1:** Studies on sexual dimorphism of the human lacrimal gland.

Author (Year)	Sample	Methodology	Main Findings and Conclusions
Waterhouse et al. (1963) [19]	Human cadaveric lacrimal glands (238 autopsy specimens: 159 females, 67 males)	Histopathological examination: Focal adenitis (inflammatory foci) in lacrimal gland tissue was identified	A higher prevalence of focal adenitis in female lacrimal glands, particularly in women over 45 years old
Damato et al. (1984) [173]	Human cadaveric lacrimal glands (99 specimens: 52 males, 47 females)	Histopathology and immunohistochemistry: Assessed acinar atrophy, periductal fibrosis, lymphocytic infiltration, and ductal changes. Investigated B lymphocyte subpopulations in 35 cases	Identified progressive acinar atrophy, fibrosis, and inflammation with age Chronic inflammation leads to ductal obstruction and lacrimal gland dysfunction No significant sex differences in B lymphocyte subpopulations
Cornell-Bell et al. (1985) [16]	Human cadaveric lacrimal glands (10 specimens: 5 males, 5 females)	Stereology, histology: Measured acinar surface area	Found a 21% difference in acinar size (larger in males: 2.18 mm^2^ vs. 1.80 mm^2^ in females) Suggested sex differences in lacrimal gland structure may be linked to androgen levels
Roen JL (1985) [164]	Human cadaveric lacrimal glands (32 specimens)	Histopathology: Examined lacrimal gland tissue for degenerative changes and inflammatory markers	Chronic inflammation and periductal fibrosis were found in 75% of samples, more common in individuals over 50. Fibrosis likely contributes to lacrimal gland dysfunction with age
Obata et al. (1995) [17]	Human cadaveric lacrimal glands (80 specimens)	Histopathology: Fibrosis, acinar atrophy, periductal fibrosis, ductal proliferation, and lymphocytic infiltration in the palpebral and orbital lobes were analysed	Diffuse fibrosis and acinar atrophy are more frequent in older women, particularly in the orbital lobe. Findings suggest these degenerative changes contribute to dry eye disease in postmenopausal women
Smith et al. (1999) [149]	Human lacrimal glands	Immunohistochemistry: Identified the location and distribution of androgen receptors in lacrimal gland tissue	ARs identified in the nuclei and cytoplasm of lacrimal acinar and ductal cells. Staining intensity and number of AR-positive cells varied among specimens. ARs are also detected in interstitial and inflammatory cells surrounding acinar units. Androgens likely play a regulatory role in lacrimal gland function. Presence of ARs in interstitial and immune cells suggests a broader role in immune modulation and glandular homeostasis
Rocha et al. (2000) [6]	Human lacrimal glands (2 specimens: 1 male, 1 female)	RT-PCR, immunohistochemistry: Analysed mRNA and protein expression of sex steroid receptors	Confirmed mRNA expression of ARs, ERs, and PRs in lacrimal gland tissue, suggesting local hormonal regulation of gland function
Wickham et al. (2000) [23]	Human lacrimal glands (3 specimens)	RT-PCR, agarose gel electrophoresis, Southern blot hybridisation: Detected mRNA for ARs, ERs, and PRs	Identified AR, ER, and PR mRNAs, suggesting lacrimal glands act as target organs for sex steroids.
Spelsberg et al. (2004) [199]	Human cadaveric lacrimal glands (20 samples from 13 cornea donors)	RT-PCR, immunohistochemistry: Detected ERs (ERα and Erβ) in lacrimal tissues.	ERα mRNA: found in 10 lacrimal gland tissue samples. ERβ mRNA: found in 9 lacrimal gland tissue samples. Postmortem time: no influence on the expression grade of ERα and ERβ. Immunohistochemical evaluation for ERα: weak staining was observed in 2 lacrimal gland samples, likely due to the thermolability of ERs and small sample sizes. Suggested potential role of estrogens in keratoconjunctivitis sicca
Lorber and Vidić (2009) [20]	Human cadaveric lacrimal glands (45 specimens: 22 males, 23 females)	Morphometric analysis: Measured lacrimal gland size and weight	Male lacrimal glands had greater absolute size and weight than female glands, suggesting structural sex differences
Gligorijević et al. (2011) [198]	Human lacrimal glands (20 specimens: 10 males, 10 females)	Immunohistochemistry: Stained for ERs and PRs	ER and PR are present in both sexes but are significantly higher in females (*p* < 0.001). The highest expression in women aged 30–50 indicates age- and sex-related differences in receptor expression.
Bukhari et al. (2014) [21]	MRI of lacrimal glands (998 lacrimal glands from 499 patients)	MRI study: Measured lacrimal gland volume across age groups, sexes, and racial backgrounds. Imaging Technique: Fat-saturated fluid-attenuated inversion recovery (FLAIR) images Volume Calculation: TeraRecon iNtuition viewer software	Lacrimal glands were larger in women. Maximum gland volume observed in the second decade of life
Hat et al. (2023) [174]	Human lacrimal glands (81 specimens: 34 females, 47 males)	Histopathology, stereology: Assessed degenerative changes and volume densities of glandular components	Acinar atrophy is significantly more prevalent in females, and ductal dilation more common in males. Females had higher fat/connective tissue volume, suggesting greater susceptibility to age-related changes
Hat et al. (2023) [163]	Human lacrimal glands (35 specimens from 19 donors: 10 females, 9 males)	qPCR: Quantification of mRNA expression for AR, ERα, and ERβ IHC Staining: Performed on selected samples to evaluate protein expression of the receptors	ERα mRNA expression was significantly higher than AR and Erβ, with no significant differences in receptor expression between sexes or correlation with age, suggesting ERα dominance in the human lacrimal gland function

RT-PCR: reverse transcriptase-polymerase chain reaction; AR: androgen receptor; ER: oestrogen receptor; PR: progesterone receptor; MRI: magnetic resonance imaging; qPCR: quantitative polymerase chain reaction; IHC: immunohistochemical.
